# Skeletal Muscle Wasting and Its Relationship With Osteoarthritis: a Mini-Review of Mechanisms and Current Interventions

**DOI:** 10.1007/s11926-019-0839-4

**Published:** 2019-06-15

**Authors:** Emily Shorter, Anthony J Sannicandro, Blandine Poulet, Katarzyna Goljanek-Whysall

**Affiliations:** 1 Institute of Ageing and Chronic Disease, University of Liverpool, William Henry Duncan Building, West Derby Road, Liverpool, L7 8TX UK; 2 Department of Physiology, School of Medicine, REMEDI, NUI Galway, Human Biology Building, University Road, Galway, Ireland

**Keywords:** Osteoarthritis, Muscle, Ageing, Exercise, microRNAs

## Abstract

**Purpose of Review:**

Osteoarthritis (OA) is a subset of joint disorders resulting in degeneration of synovial joints. This leads to pain, disability and loss of independence. Knee and hip OA are extremely prevalent, and their occurrence increases with ageing. Similarly, loss of muscle mass and function, sarcopenia, occurs during ageing.

**Recent Findings:**

Little is known about the impact of muscle wasting on OA progression; nevertheless, it has been suggested that muscle wasting directly affects the stability of the joints and loss of mobility leads to gradual degeneration of articular cartilage. The molecular mechanisms underlying muscle wasting in OA are not well understood; however, these are probably related to changes in gene expression, as well as epigenetic modifications.

**Summary:**

It is becoming clear that skeletal muscle wasting plays an important role in OA development and/or progression. Here, we discuss mechanisms, current interventions, such as exercise, and potentially novel approaches, such as modulation of microRNAs, aiming at ameliorating OA symptoms through maintaining muscle mass and function.

## Introduction

Osteoarthritis (OA) is a multifactorial condition associated with degeneration of the joint, with pathological changes to multiple musculoskeletal tissues such as cartilage, meniscus, ligament and synovium. OA is characterised by degradation of articular cartilage, subchondral bone sclerosis, the formation of osteophytes at the joint margin and synovitis [1]. Synovial inflammation (synovitis) has been demonstrated during all stages of OA with synovium showing thickening and inflammation even before visible cartilage degeneration [2]. In addition, anterior cruciate ligament (ACL) injury, especially with concomitant ligament or meniscal pathology, increases the risk of knee OA suggesting an important role of healthy ligaments in maintaining joint tissue homeostasis [3]. OA patients and rodent models of OA are characterised by changes in cartilage, meniscus, synovium and ligament, suggesting common mechanisms of joint degeneration during OA development [1, 4]. Therefore, OA can be thought of as an organ failure of the whole synovial joint. Linked to the joint, muscles also play a major role in joint function during movement and in joint stability, but this link has been seldom explored. In this mini-review, we aim to provide current information linking muscle wasting and OA.

## Muscle Wasting in OA

The progressive loss of periarticular muscle mass and function has consequences on joint stability and health. Muscle wasting is inevitably associated with ageing, and, more recently, it has been demonstrated in patients with OA [5–7]. The reduction in muscle mass and strength is attributed to myofibre atrophy, reduction of muscle quality and defective muscle regeneration (reviewed in [8]). Until recently, research investigating the association of muscle wasting with OA has been lacking. Currently, more and more data support the relationship between joint health and the surrounding skeletal muscle; however, functional and mechanistic studies are still lacking.

Muscle contractility is required for joint formation already during embryogenesis [9], and muscle weakness is an important determinant of pain and disability during OA [10]. Several studies have shown that a decrease in lower limb lean mass is frequent in OA patients [11], and this is associated with a greater risk of falls [12–14]. Progressive muscle weakness in OA is also associated with muscle fibre atrophy, with studies demonstrating 12–19% reduction in cross-sectional area of muscles in patients with hip and knee OA [6]. Moreover, muscle damage is associated with articular cartilage degeneration [15], whereas the increased cross-sectional area of v*astus medialis* is associated with improved structural changes at the knee and reduced pain in OA patients [16].

On the other hand, a large longitudinal cohort study found that, in 1653 subjects without radiographic knee OA (ROA) at baseline, an increased risk of ROA was not associated with sarcopenia alone, but rather with sarcopenic and body composition–based obesity [17]. Though, it should be noted that this study classified sarcopenia as the sum of absolute muscle mass of upper and lower limbs. However, findings from a previous study suggest that skeletal muscle mass of the lower limbs shows a higher correlation with knee OA than that of the lower and upper limbs combined [18]. Therefore, a statistical association between the risk of knee OA and sarcopenia may be observed, if the assessment of skeletal muscle mass focuses on the lower limbs.

A study investigating the influence of pain and ROA on muscle mass, strength, quality and risk of falls in older adults showed that hip and knee ROA are not significantly associated with changes in muscle strength and quality, despite the association of self­reported lower extremity joint pain, stiffness and dysfunction with declines in the aforementioned muscle parameters in older women. As such, it was concluded that pain may be the underlying mechanism via which OA leads to functional decline [19].

These contradictory results may be associated with a lack of a clear definition of sarcopenia. In recent years, many definitions of sarcopenia have been proposed, each one recommending diagnostic criteria based on muscle mass combined with measures of muscle strength, function or physical performance [20]. It is therefore crucial that criteria for the definition of sarcopenia are established and adopted in order for research to obtain results that are clinically relevant.

Another limitation of research in this area is the lack of a clear method to correlate the development of OA and incidence of sarcopenia. The current method used by research to investigate this relationship is to separately assess the OA severity grade and the lean body mass (LBM) of the area of interest [21]. Moreover, most of the research into OA and sarcopenia focuses on whether or not there is a correlation between the two disorders and has yet to fully investigate the molecular mechanisms behind the observed changes. It has been suggested that myokines, muscle-produced cytokines, peptides and growth factors communicate with the surrounding articular components such as the synovium, cartilage and bone through paracrine mediation, and thus may affect the signalling cascades implicated in OA [22].

Of the studies exploring molecular mechanisms underlying muscle wasting in OA, most have focused on inflammatory mediators as the molecular link between muscle function and OA. For example, Levinger et al. [23] observed increased inflammation in the *vastus lateralis* in patients with knee OA compared with a control group, based on increased protein abundance of p65 NF-κB, STAT-3 and JNK. However, a recent study attempted to investigate changes in muscle quality in OA by conducting the first cellular-level analysis of the *vastus lateralis* in adults with moderate knee OA [24]. Results of this study showed significant pathogenic fibrosis in the muscle of OA patients. Moreover, aberrant collagen deposition was noted in the extracellular matrix of OA muscle, which was significantly associated with decreased satellite cell density, as well as muscle strength. Increased expression levels of both CCN2 and TGFβ mRNAs were correlated positively to the amount of collagen deposition and inversely correlated with muscle strength. Finally, results indicated a muscle fibre type shift in the OA group, with significantly more type IIa/x hybrid fibres and fewer type I fibres in OA muscles relative to controls. Despite the limitations of this study, including low sample sizes and its cross-sectional nature, the data provide excellent molecular insight into the pathology of muscle changes that occur during OA. Nonetheless, there remains a large insufficiency of research in this area, making it essential that future studies focus on elucidating the mechanisms behind the influence of OA on skeletal muscle in order to develop more targeted therapeutic approaches. Interestingly, we have also shown that, in a spontaneous model of OA (STR/Ort mice), significant changes occur in the expression of muscle-related genes in the articular cartilage, suggesting at least some common molecular mechanisms underlying degeneration of musculoskeletal tissues during OA [25].

Another question remains to be answered: whether muscle changes precede OA, or vice versa. Studies have shown that low muscle quality and the depletion of lean body mass of lower extremities/body weight may be risk factors as important a contributor to OA pathogenesis as mechanical influences [26]. On the other hand, it has been suggested that the disuse of an OA affected joint, due to the pain of movement, may be the primary cause of the reduction in muscle strength associated with OA [27]. To add to the complexity, data from a study investigating muscle wasting in an anterior cruciate ligament transection (ACLT) model of OA suggest that disuse does not fully explain the muscle wasting observed in OA patients [6]. Results of this research show that rats in the OA group display the same pattern of movement as those of SHAM group (i.e., rats submitted to surgery without ALCT). Despite the similar locomotion (measured as spontaneous exploratory velocity and distance), the *gastrocnemius* cross­sectional area was reduced by approximately 10% in the OA group. It was concluded, therefore, that muscle wasting may be a consequence of chronic, low-grade inflammation associated with OA, rather than solely joint disuse. However, the authors did acknowledge that the rats in the OA group displayed significantly increased nociception towards the end of the study, suggesting that the extended experimental period could have allowed for the detection of reduction in joint use.

The results of the above studies highlight the need to monitor the tendency of OA patients to avoid physical activity. It may prove beneficial for patients identified from this assessment to undergo therapeutic exercise intervention as a means to alleviate the symptoms of OA, as well as to improve their muscle strength.

## Exercise Interventions to Maintain Muscle Mass and Function During OA

Current exercise interventions for OA focus highly on lifestyle modification, weight reduction and specific exercise interventions that focus on strengthening periarticular musculature [28, 29]. This is not without challenge, however, as many OA patients are unable to tolerate the load intensity required for progressive overloading of the relevant musculature [28]. Fortunately, the examination of modifications in low impact exercise, such as swimming and cycling, has shown a beneficial reduction in pain, stiffness and functional deficits in patients with knee OA [29, 30]. In a 12-week study examining swimming and cycling as exercise therapy for moderate knee OA, patients from both groups showed increased distance achieved in a 6­minute walk test, as well as increased isokinetic knee extensor and flexor strength. Additionally, in the swimming cohort, there was an approximately 40% reduction in joint pain, as assessed by the Western Ontario and McMaster Universities Osteoarthritis Index (WOMAC) [29]. Limitations to this study, however, were the lack of participation of patients with advanced OA and the short intervention time. Other areas of research have evaluated similar low-load (20­50% of one-repetition maximum) exercise training in knee OA patients utilising a partial vascular occlusion approach, wherein a pressure cuff or tourniquet is applied to the upper thigh, with similar results in pain reduction and functional improvements as compared with the conventional strength-training group (i.e., 70% of one-repetition maximum), yet with reduced knee pain whilst exercising [28]. The assumption is that this technique provides greater type II fibre activation via a generated anaerobic environment, whilst other possible mechanisms include increased intracellular metabolites (e.g., H+ protons, lactate and adenosine monophosphate), which in turn may stimulate growth hormone secretion [28, 31].

The majority of the literature suggests that sedentary lifestyle behaviours and immobilization are associated with cartilage disease and progression of OA, whereas controlled loading of the knee is associated with favourable changes to the extracellular matrix and molecular biomarkers associated with OA such as C­reactive protein and IL-10 [32]. Therapeutic exercise is beneficial to cartilage health, as the literature suggests molecular biomarkers associated with inflammation (i.e., CRP and TNFα) and cartilage breakdown (C12C, CTX-II and COMP) are either unchanged or downregulated in studies examining these characteristics in exercise and OA [32]. Furthermore, exercise programmes focusing on neuromuscular control, such as closed chain kinetic and proprioceptive exercises, are generally well tolerated in OA patients and show results similar to more traditional strengthening exercises [33, 34]. This is consistent with literature suggesting that aberrations in the neuro-motor system are involved in changes to muscle force patterns, with altered joint reaction forces leading to focal alterations to cartilage loading over time [35]. This suggests that there is a complex relationship between both muscular strength and changes to motor unit physiology in OA, and strengthening alone may not be the only consideration in clinical practice [35]. Meta-analyses show that no specific protocol is deemed the best and current guidelines are ambiguous in recommending exercise programs [36]; thus, the best exercise programme may be the one that is met with high adherence by the individual and tolerated enough to provide consistent progression over time with graded adjustments to load and volume. Recent exercise interventions in OA patients are summarised in Table 1.

**Table 1:** Summary of research examining effects of exercise on muscle during osteoarthritis

Exercise type	Sex	Effects	References
Moderate intensity (60% of one-­repetition maximum) resistance exercise	Males and females with knee OA	•Increased quadriceps CSA ~ 7% • No change in single- muscle fibre CSA • Increased intermyofibrillar mitochondrial size in men • Improved one-repetition maximum strength • Improved contractility in men • Minimal effects to improve muscle atrophy	[35]
Neuromuscular exercise or quadriceps strengthening	Males and females with knee OA	• Both neuromuscular exercise (NEMEX) and quadriceps strengthening groups showed improvements in pain and function • No observed differences were seen between either NEMEX or quadriceps-specific strengthening with respect to strength improvements	[33]
Resistance training with partial vascular occlusion (PVO) (30% of one- repetition maximum)	Females with knee OA	• Reduced anterior knee pain with PVO compared with conventional loads (70% one-­repetition maximum) • Both PVO and conventional load groups showed improvements in quadriceps strength, function and pain	[28]
Resistance based circuit training	Males and Females with knee OA	• Increased walking speed • Reduction in pain • Increased strength of knee flexors and extensors • Increased isometric strength of hip abductors	[30]
Non-­weight bearing resistance exercise (swimming/cycling)	Females with knee OA	• All groups reported reductions in pain, joint stiffness and physical limitation • Increased function assessed by hand grip, knee extension and flexion power and increased distance in 6-­min walk test • Do differences observed between groups (swimming vs cycling)	[29]
Aerobics/step- aerobics	Females with knee OA	• Improved cartilage quality • 11% greater isometric knee extension force compared with control • No difference in self-rated knee OA symptoms	[37]

## microRNAs as Novel Therapeutic Targets for Muscle Wasting During OA

MicroRNAs (also referred to as miRNAs or miRs) are a class of small non-coding RNA molecules, approximately 22 nucleotides long, which bind to their target messenger RNAs (mRNAs), induce mRNA degradation or inhibit protein translation, and thus exert post-­transcriptional regulation of gene expression [38]. Studies report that specific miRNA-target interactions may regulate all major known contributors to the onset of the senescent phenotype, such as DNA damage, telomere shortening, protein misfolding and oxidative stress [39]. Therefore, recent research has focused on investigating whether restoring physiologic levels of specific miRNAs in these tissues can restore—at least partially—functional homeostasis [40]. Investigating the use of miRNA therapies in disorders such as OA is particularly important given that the only current treatment options for the condition are pain and symptom management and eventual joint replacement therapy [41]. It is anticipated that elucidating the underlying molecular mechanisms of the disorder, in particular the associated genetic and epigenetic dysregulation, will lead to the development of novel interventions to delay the need for invasive and transient total joint replacement procedures.

One of the most well-studied miRNAs in OA research, to date, is miR-140, a cartilage-specific miRNA [42], which was first implicated in OA pathogenesis in a study by Miyaki et al. This study showed that miR-140 expression in articular cartilage from OA donors is significantly lower than that in normal cartilage or following treatment with IL-1β, a cytokine known to be involved in the pathogenesis of OA. Interestingly, the transfection of chondrocytes with miR-140 downregulated IL-1β-induced ADAMTS5 gene expression. The correlation of abnormal miR-140 expression in OA cartilage with increased ADAMTS5, as first reported in this study [42], suggests that miR-140 may play an important role in maintaining cartilage homeostasis. Since then, multiple studies have shown that miRNAs play significant roles in cartilage homeostasis. Moreover, there is evidence that miRNAs are important players in maintaining the health of all joint tissues, including muscle [43–47], cartilage [48, 49] and ligament and tendon [50, 51]. Whilst there is very little known about the role of specific microRNAs in muscle wasting during OA, with only one microRNA, miR-141, suggested to have a function in the ageing of the multiple tissues of the musculoskeletal system [52], multiple studies have shown the involvement of miRNAs in muscle wasting [53–55]. The importance of microRNAs in the function of musculoskeletal tissues was also demonstrated in depletion studies of Dicer, a key enzyme in miRNA biogenesis. The deletion of Dicer in chondrocytes resulted in defective cell proliferation and differentiation [51], and the deletion of Dicer in bone progenitors was associated with defective bone formation [56]. A satellite cell–specific Dicer knockout resulted in mild muscle fibre atrophy over time [57], and microRNAs maintain muscle homeostasis through the control of muscle hypertrophy and atrophy and muscle adaptation to exercise [58–60]. Below, we focus on several examples of microRNAs with a potential role in regulating homeostasis of muscle and other joint tissues in the context of OA.

miR-143 is differentially expressed in the cartilage of older people suffering from OA [53]. Interestingly, miR-143 regulates the phenotype of synovial fibroblasts in rheumatoid arthritis through the regulation of IGFBP5 [61]. Our group has previously demonstrated the regulation of senescence of satellite cells, adult skeletal muscle stem cells, via the miR-143:Igfbp5 regulatory network [47]. Interestingly, miR-­143:Igfbp5 interactions are regulated by inflammatory cytokines, including IL6.

Our group has also shown that miR-181 is downregulated in muscle during ageing and regulates the expression of Sirt1 [46]. Interestingly, another group has shown that the expression of miR-181 is also altered in the joints of OA patients and that inhibition of this microRNA in animal models of OA attenuates OA in facet and knee joints [62].

Another microRNA downregulated in muscle of humans during ageing is miR-378 [63], which controls myogenesis, metabolism and autophagy [55, 64, 65]. Indeed, molecules regulating miR-378 have been patented for the use in regulating metabolism in different tissues, including skeletal muscle (patent number WO2011153542A3). The changes in the regulation of cellular metabolism, for example through autophagy, have also been noted during OA, and miR-378 has been detected in synovium in late-stage OA patients [66, 67]. Moreover, miR-378 promotes osteogenesis in human bone marrow–derived mesenchymal stem cells, suggesting that it is a pro-bone regeneration molecule [68]. Together, these data indicate a potential common mechanism, via miR-378 and other microRNAs, underlying musculoskeletal tissues homeostasis.

Whilst multiple studies have focused on the role of microRNAs in muscle development and regeneration, very few functional studies exist that have characterised the role of microRNAs in muscle wasting during ageing or OA [69, 70].

It also has to be noted that Soares et al. [45] demonstrated that microRNA function is context dependent in different models of muscle atrophy; therefore, a question arises whether the miR-associated mechanisms of muscle wasting during ageing are similar or different to those observed during muscle wasting in OA. Functional studies of microRNAs in muscle during OA are needed.

## Conclusions

OA is a progressive and debilitating disease and the most common cause of chronic disability in adults. Despite this, pain management and total joint replacement procedures are the only current treatment options for the disease. As such, a lot of research into OA has focused on the development of novel interventions to delay the need for invasive total joint replacement procedures. The association between skeletal muscle mass and function and OA development and progression remains to be elucidated; however, it is becoming clearer that changes in skeletal muscle are important players in OA. As such, exercise interventions could benefit OA patients not only due to benefits on muscle tissues, but also through all joint tissues affected by OA. An emerging approach to interventions against OA includes the regulation of expression and function of microRNAs. Although the role of microRNAs in muscle wasting in OA is not yet understood, given that a single microRNA can regulate multiple genes and dysregulation of microRNA expression occurs in a variety of pathological conditions, microRNAs are emerging powerful regulatory molecules and potential novel therapeutic agents; indeed, microRNA­based therapies are currently undergoing clinical trials [71].

Though currently in its infancy, the use of miRNA-based therapeutics in OA, including but not limited to regulating muscle mass and function, is a promising avenue of research, with many recent studies suggesting that their use may overcome the limitations of many traditional therapies. However, functional approaches are lacking. Moreover, experiments using more relevant animal OA models and large-scale or cohort-based clinical trials should be conducted in order to comprehensively evaluate the efficacy of miRNA therapeutics in OA.
